# Temporal interference stimulation targeting right frontoparietal areas enhances working memory in healthy individuals

**DOI:** 10.3389/fnhum.2022.918470

**Published:** 2022-10-28

**Authors:** Yufeng Zhang, Zhining Zhou, Junhong Zhou, Zhenyu Qian, Jiaojiao Lü, Lu Li, Yu Liu

**Affiliations:** ^1^Key Laboratory of Exercise and Health Sciences of Ministry of Education, Shanghai University of Sport, Shanghai, China; ^2^Hebrew SeniorLife, Hinda and Arthur Marcus Institute for Aging Research and Harvard Medical School, Boston, MA, United States

**Keywords:** temporal interference stimulation, healthy human participants, working memory, transcranial alternating current stimulation, cognition

## Abstract

**Background:**

Temporal interference (TI) stimulation is a novel technique that enables the non-invasive modulation of deep brain regions. However, the implementation of this technology in humans has not been well-characterized or examined, including its safety and feasibility.

**Objective:**

We aimed to examine the feasibility, safety, and blinding of using TI on human participants in this pilot study.

**Materials and methods:**

In a randomized, single-blinded, and sham-controlled pilot study, healthy young participants were randomly divided into four groups [TI and transcranial alternating current stimulation (tACS) targeting the right frontoparietal region, TI-sham, and tACS-sham]. Each participant was asked to complete N-back (*N* = 1 to 3) tasks before, during, and after one session of stimulation to assess their working memory (WM). The side effects and blinding efficacy were carefully assessed. The accuracy, reaction time (RT), and inverse efficiency score (IES, reaction time/accuracy) of the N-back tasks were measured.

**Results:**

No severe side effects were reported. Only mild-to-moderate side effects were observed in those who received TI, which was similar to those observed in participants receiving tACS. The blinding efficacy was excellent, and there was no correlation between the severity of the reported side effects and the predicted type of stimulation that the participants received. WM appeared to be only marginally improved by TI compared to tACS-sham, and this improvement was only observed under high-load cognitive tasks. WM seemed to have improved a little in the TI-sham group. However, it was not observed significant differences between TI and TI-sham or TI and tACS in all N-back tests.

**Conclusion:**

Our pilot study suggests that TI is a promising technique that can be safely implemented in human participants. Studies are warranted to confirm the findings of this study and to further examine the effects of TI-sham stimulation as well as the effects of TI on deeper brain regions.

## Introduction

Maintaining brain health is critical to multiple functions in human beings. The advancement of neuromodulation techniques has enabled the modulation (i.e., facilitation or inhibition) of the excitability of brain regions/networks, thus benefiting disease therapy and the rehabilitation of functions in different populations (Summers et al., 2016; Reinhart and Nguyen, 2019; Ma et al., 2020). In contrast with invasive brain stimulation techniques, such as deep brain stimulation using *in vivo* electrodes *via* surgery to stimulate brain regions (Hacker et al., 2018; Huh et al., 2018), non-invasive brain stimulation (NIBS) techniques, including transcranial electrical [i.e., direct (tDCS), alternating (tACS), and random noise (tRNS)] stimulation (tES), modulate the cortical activities of the brain using non-invasive scalp electrodes to deliver the currents without the need for surgery (Fertonani and Miniussi, 2017; Elyamany et al., 2021). An increasing number of research efforts have been focused on NIBS technology, as these techniques induce improvements in functions (e.g., cognitive and motor function) with mild or no side effects (Reinhart and Nguyen, 2019; Guo et al., 2020; Pilloni et al., 2020). However, modeling studies have shown that the electric field generated by tES usually drops quickly as the depth of stimulation increases due to the high electrical resistivity of the head (Datta et al., 2009). Increasing the current intensity can help resolve these issues, which, on the contrary, can cause severe safety issues (Antal et al., 2017). Moreover, the focality of tES is limited, especially in deep brain regions, due to the diffusion of current flow (Datta et al., 2009; Lee et al., 2020). Therefore, the application of tES techniques has mostly focused only on the cortical regions of the brain (Schönfeld and Wojtecki, 2019), largely limiting their implementation to stimulating subcortical regions (e.g., basal ganglia) (Lozano et al., 2019; Schönfeld and Wojtecki, 2019).

Recently, a novel non-invasive technique called temporal interference (TI) stimulation was developed, and this technique has shown that it is able to overcome some limitations of the traditional tES techniques (Grossman et al., 2017). Specifically, this TI technique uses scalp electrodes to simultaneously deliver two high-frequency electrical sinusoidal waveforms, with a frequency difference between each electrode pair. These two waveforms can then generate an amplitude-modulated waveform at a slower frequency in the target regions (Grossman et al., 2017). For example, when sinusoidal waves of 2,000 and 2,010 Hz were superimposed, a 10 Hz amplitude-modulated waveform was generated (Grossman et al., 2017). These generated electric fields are vectors that are summed to produce the strongest effect in the target brain region. It should be noted that the target location of the modulation can be altered by adjusting the electrode placement and current settings for TI stimulation (Grossman et al., 2017). Furthermore, the target depth of stimulation gets deeper as the electrode spacing widens (Grossman et al., 2017). Animal studies have been completed to test the effects of TI on brain activity, with the results showing that TI is a promising strategy to induce the expected changes within the targeted regions (Grossman et al., 2017; Lee et al., 2020). To date, only two studies have explored the effects of TI stimulation on human participants (Ma et al., 2021; Zhu et al., 2022), showing promising TI-targeting results in the motor cortex of the brain. However, inappropriate statistical methods (i.e., *post-hoc* analysis with non-significant effects on the slope of the IO curve) (Ma et al., 2021) and a sham control design (i.e., sham-controlled protocols with tDCS) (Zhu et al., 2022) limited the power of their findings. Furthermore, only Ma et al. (2021) evaluated side effects, and neither study assessed the blinding efficacy (Zhu et al., 2022). As a result, more TI stimulation studies are necessary to characterize the side effects and blinding efficacy in humans, which are critical to the implementation of this technique in future research.

We thus conducted a randomized, single-blinded, and sham-controlled pilot study to explicitly examine the feasibility of implementing TI in human participants. The primary goal of this study was to characterize the blinding efficacy and potential side effects of TI targeting the frontoparietal regions of healthy younger adults. We also examined the effects of TI targeting in the frontoparietal regions on working memory (WM). Recent evidence has shown that the regulation of WM is dependent upon its activation within the frontoparietal regions of the brain, and using tACS to target this region may help to enhance WM (Violante et al., 2017). Thus, we implemented TI with the same frequency as tACS that has been found to be beneficial to WM and examined whether the effects induced by TI were comparable to those induced by tACS. Specifically, we hypothesized that (1) TI would not induce moderate-to-severe side effects; (2) TI blinding would be successful; and (3) compared to the sham group, TI targeting of the frontoparietal regions would induce significant improvements in WM, and such improvement would be comparable to that induced by tACS targeting the same region.

## Materials and methods

### Participants

Sixty healthy young adults (26 males and 34 females) between the ages of 18 and 28 were recruited to participate in this study. The inclusion criteria were as follows: (1) right-handed and (2) no history of psychiatric or neurological disorders (e.g., stroke) or drug abuse. The exclusion criteria were as follows: (1) cognitive impairment as assessed by Mini-mental State Examination (MMSE) score <27; (2) participation in other studies using NIBS within 1 week; (3) any medication or psychotropic drugs used in the 4 weeks before the study; (4) legal blindness; (5) inability to follow the instructions to complete the study; (6) the presence of metal implants. All participants who were recruited met the inclusion and exclusion criteria. The study protocol was approved by the Institutional Review Board of the Shanghai University of Sport, China (approval number: 10277202112T096). Participants provided written informed consent to participate in the study.

### Design and procedure

**FIGURE 1:**
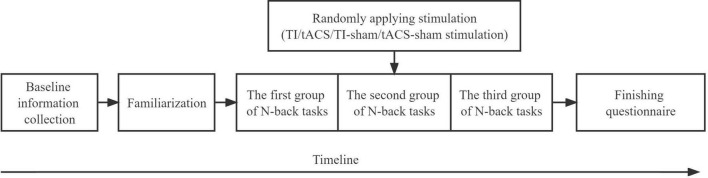
Study procedure. TI, temporal interference; tACS, transcranial alternating current stimulation.

For the randomized controlled and single-blinded pilot study, the 60 participants were randomly assigned into three groups, including the TI group (*n* = 20, age = 22.60 ± 2.54 years), tACS group (*n* = 20, age = 22.45 ± 2.52 years), and sham group (*n* = 20, age = 22.2 ± 2.35). Because the devices used to apply TI and tACS were different, the sham group was divided into two groups: TI-sham group (*n* = 10, age = 20.50 ± 2.12 years) and tACS-sham group (*n* = 10, age = 23.90 ± 0.87 years). Therefore, four groups were included in the current study. The participants were blinded to the actual stimulation protocol they received. The randomization of the stimulation group was completed using a Latin Square Design. Within 24 h before the visit, participants were required to avoid high-intensity physical activity; drinking caffeinated drinks, strong tea, and alcohol; and smoking cigarettes. During the stimulation visit, the participants first practiced the N-back tasks to ensure that they were familiar with the task procedure. After familiarization, each participant completed tasks consisting of 1, 2, and 3 backs before, during, and immediately after the stimulation session (pre-, during-, and post-stimulation) (Polanía et al., 2012; Röhner et al., 2018). By the end of the visit, the participants were required to complete questionnaires to assess the blinding efficacy and side effects (Figure 1).

### N-back tasks assessing working memory

We used the Psychtoolbox in MATLAB (MathWorks, Natick, MA, United States) for the N-back tasks, and the tasks were displayed on a monitor in front of the participants. These tasks had three conditions: 1-back, 2-back, and 3-back. Each participant completed one group of N-back tasks pre-, during-, and post-stimulation. Each group of tasks consisted of nine blocks (i.e., three blocks per condition). Participants had the opportunity to rest between the blocks for 30 s, which was self-paced (i.e., could have been shorter). Each block comprised 40 trials (i.e., 40 × 9 = 360 trials in nine blocks), and the order of the blocks was randomized. Within each trial, participants were presented with a single letter (A–Z) sequentially. Each letter (Arial font style, height at a visual angle of 2 degrees) was displayed for 300 ms followed by a fixation cross (size = 1 × 1 degrees of visual angle) for 1,700 ms. During the 1-back task, participants were asked to detect whether the stimulus matched the one presented previously. In the 2-back and 3-back tasks, the participants detected the repetition of the letter shown two or three trials prior. Target trials occurred in 40% of the trials. Participants were instructed to press the “1” button on a keyboard using their right hand or to press the “2” button if the current stimulus matched the stimulus presented in N items that were seen previously or if the stimulus did not match, respectively, as quickly and accurately as possible.

We quantified the reaction time [RT, millisecond (ms)], accuracy, inverse efficiency score (IES), and accuracy-weighted RT value (i.e., RT/accuracy) (Reeder et al., 2017), of each response. We only included the RT of the correct responses in the analysis. Because of the significant difference in the accuracy and IES before stimulation between groups (see Table 1), the percent change for each outcome variable from pre- to during-stimulation (percent change 1) as well as from pre- to post-stimulation (percent change 2) was calculated and used in the following analysis to assess the performance on the N-back tasks and was expressed as the mean ± standard error: percent change 1 = [(pre-stimulation values)–(during-stimulation values)]/(pre-stimulation values) and percent change 2 = [(pre-stimulation values)–(post-stimulation values)]/(pre-stimulation values). The RT, accuracy, and IES of the 3-back task were used as our primary outcome variables, and the RT, accuracy, and IES of the 1-back and 2-back tasks were our secondary outcome variables.

**TABLE 1:** The baseline characteristics of four groups (mean ± SD).

Variables	TI (*n* = 18)	tACS (*n* = 18)	TI-sham (*n* = 8)	tACS-sham (*n* = 10)	*F*	*P*
Age, years	22.39 ± 2.81	22.61 ± 2.45	20.00 ± 0.92	23.90 ± 0.87	4.635	0.006*
Female, *n* (%)	11 (61.1%)	9 (50.00%)	4 (50.00%)	6 (60.00%)		0.89
Education, years	16.06 ± 2.48	15.44 ± 1.82	13.38 ± 0.51	17.10 ± 0.31	6.716	0.001*
ACC 1-back	0.96 ± 0.03	0.96 ± 0.04	0.95 ± 0.02	0.95 ± 0.03	0.519	0.671
RT 1-back	0.61 ± 0.12	0.64 ± 0.11	0.72 ± 0.16	0.66 ± 0.12	1.505	0.225
IES 1-back	0.64 ± 0.11	0.67 ± 0.11	0.76 ± 0.18	0.70 ± 0.13	1.763	0.166
ACC 2-back	0.93 ± 0.04	0.91 ± 0.06	0.93 ± 0.05	0.90 ± 0.05	0.93	0.433
RT 2-back	0.77 ± 0.19	0.81 ± 0.15	0.80 ± 0.14	0.82 ± 0.14	0.273	0.844
IES 2-back	0.83 ± 0.20	0.90 ± 0.19	0.88 ± 0.18	0.91 ± 0.20	0.607	0.613
ACC 3-back	0.88 ± 0.08	0.81 ± 0.08	0.89 ± 0.10	0.80 ± 0.10	3.613	0.019*
RT 3-back	0.803 ± 0.16	0.886 ± 0.15	0.815 ± 0.17	0.835 ± 0.14	0.967	0.416
IES 3-back	0.909 ± 0.17	1.101 ± 0.20	0.938 ± 0.28	1.061 ± 0.26	2.837	0.047*

1-back, 2-back, and 3-back, three types of N-back tests; TI, temporal interference; tACS, transcranial alternating current stimulation; ACC, accuracy; RT, reaction time; IES, inverse efficiency score. F-value represents the F-test statistic from the ANOVA. P-value represents the difference between groups; the * sign indicates a significant difference.

### Stimulation protocols

#### Temporal interference stimulation

**FIGURE 2:**
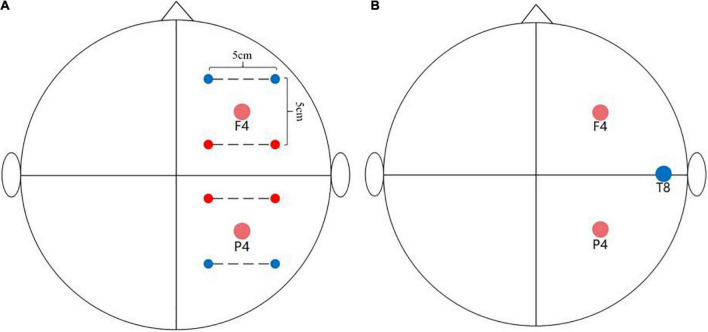
Stimulation montage. **(A)** TI stimulation montage: The two red dots and two blue dots are connected by the dotted line representing the alternating currents of 2,000 and 2,006 Hz, respectively. The distance between the center of each electrode was 5 cm. **(B)** tACS stimulation montage. The two red dots represent the position of the two stimulation electrodes and the blue dot represents the position of the return electrode.

The design of the temporal interference stimulation system (TIESS) was based on the circuit shown in Grossman et al.’s (2017) study (Supplementary Figure 1), and the stability of the output current was also examined (Supplementary Figures 2, 3). Specifically, the system is able to program and send the alternating currents *via* four channels (four pair of electrodes). In this study, the frequency of the currents was set at 2,000 Hz for two channels and 2,006 Hz for the other two channels. The peak-to-peak amplitude of the current was 2 mA for each pair of electrodes, so the maximum current of the envelope signal delivered to the target region was 4 mA. The stimulation target of this study was the middle frontal gyrus and inferior parietal lobule, that is, F4 and P4 of the EEG 10/20 system. To achieve this, ring electrodes (Soterix, America) were placed on the scalp and were parallel to each target with, a distance of 5 cm between the centers of each electrode (Figure 2A). The frequency difference (Δf) on each target was 6 Hz (Δf = 2,006–2,000 Hz). The frequency of 6 Hz was used in this study because many studies have previously found that theta tACS can regulate the WM process (Violante et al., 2017; Bender et al., 2019). Because we adopted an online stimulation design, when the participants began to perform the second group of N-back tasks, stimulation was applied. When the second group was completed, stimulation was stopped (Polanía et al., 2012). In total, the stimulation lasted for about 15 min. The stimulation began with a 15 s ramp-up period.

#### Electromagnetic computation

The numerical calculations were performed by solving the equation ∇⁡σ∇⁡ϕ = 0, where σ is the electrical conductivity, and φ is the electric potential. The current density was obtained through E = −∇⁡ϕ and j =σ⋅E. The displacement component of the current was neglected, and the same value of conductivity was used under all frequencies, as in previous studies (Hasgall et al., 2015; Grossman et al., 2017). The cylindrical phantom had a dielectric inside and a vacuum on the outside, and each region was treated as having uniform conductivity. The equation was solved numerically by the eigenfunction expansion method by matching the boundary conditions. The stimulating currents were normalized by integrating the total current density. The envelope modulation amplitude distribution of the TI electric field in *x*- or *y*-directions was obtained by computing the difference between the peak and through the envelope wave caused by TI in the specific orientation. This electromagnetic computation aimed to compare the distribution properties and spatial gradients in the TI and tACS fields.

#### Transcranial alternating current stimulation

Transcranial alternating current stimulation was delivered by the NeuroConn system (DC-STIMULATOR, NeuroConn Inc., Ilmenau, Germany). The saline-soaked sponge electrodes (5 cm × 5 cm) were placed at F4 and P4, and the return electrode was placed at T8 (Figure 2B) of the EEG 10–20 system. The parameters of tACS were similar to those used in TI, that is, sinusoidal currents with a 6 Hz frequency value were used. No phase offset was observed between the two circuits, and the peak-to-peak amplitude was 2 mA without DC offset. The stimulation began with a 15 s ramp-up and lasted approximately 15 min (Violante et al., 2017; Bender et al., 2019).

#### Sham stimulation

Since we used two types of stimulation, we also designed two types of sham stimulation as the control (i.e., TI-sham and tACS-sham). For TI-sham, we used the TIESS, as described above. For tACS-sham, we used the same NeuroConn system as the one used for tACS. The montages of the two types of sham stimulation were the same as those used in TI and tACS, respectively. Specifically, the number of electrodes and the placement of the electrodes for the TI-sham were the same as those used in TI stimulation. Likewise, the number of electrodes and the placement of the electrodes for the tACS-sham were the same as those used in tACS. The sham condition only consisted of a ramping up stage and a fade-out stage. Both stages lasted 15 s.

### Blinding efficacy and side effects

To assess the blinding efficacy, all participants were asked to guess the type of stimulation they received (i.e., real stimulation, sham, or uncertain) at the end of the visit. To assess the potential side effects, they were also asked to report whether any NIBS-related side effects were experienced during the stimulation period *via* the side-effect questionnaire. Side effects included pain, itching, burning, skin redness, and fatigue, and the severity of each sensation could be rated as none (0), mild (1), moderate (2), or severe (3). In addition, to avoid ignoring other side effects, we asked open questions at the end of the questionnaire to determine whether the participants experienced any other discomfort. The detailed side-effect questionnaire is included in Supplementary material.

### Statistical analysis

Statistical analyses were performed using IBM SPSS Statistics 24 and JMP Pro 14 software (SAS Institute, Cary, NC, United States). The Shapiro–Wilk test was used to test the normal distribution of the outcome variables. The significance level was set at *p* < 0.05. Baseline characteristics were analyzed by one-way analysis of variance (ANOVA) for continuous variables and Fisher’s exact test for dichotomous variables.

Fisher’s exact test was used to examine the blinding efficacy. One-way ANOVA was used to examine the effects of TI on the performance of the N-back tasks (both the primary and secondary outcome variables). ANOVA was used with four levels (TI, tACS, TI-sham, and tACS-sham) of the stimulation model factors. The dependent variable was the percent change of the outcome variables (RT, accuracy, and IES) obtained from the N-back test. Tukey’s *post-hoc* analysis was used if significance was observed in the ANOVA models. Age and education were used as the covariates in the ANOVA tests. When there was a significant difference between groups in the outcome as measured pre-stimulation, the ANOVA model was adjusted for that pre-stimulation value. When appropriate, the *P*-values and *F*-values representing the *F*-test statistic of the ANOVA were quoted.

To examine the side effects induced by stimulation, Kruskal–Wallis tests were used to compare ranked categorical variables described as numbers: side effects (e.g., pain, itchiness, and burning) as reported by participants in the four groups based on information from the side-effect questionnaire. Kendall’s tau non-parametric correlations were used to explore the relationships between the severity of the reported side effects and the guessed stimulation type that the participants received. To examine the potential effects of the participant being able to guess of the stimulation condition on their performance, separate ANOVA models were used within the TI groups. The participant’s guess (i.e., real, sham, or uncertain) was used as the model factor, and the dependent variables were the percent change of the outcome variables, which were significantly improved by TI, from pre- to post-stimulation.

## Results

Sixty eligible younger adults participated in this study. Six were removed from the study due to the failure to complete the N-back tests (e.g., accuracy lower than 75%) (Violante et al., 2017). Therefore, data for 54 participants were used in the analysis (Table 1).

Age (*F* = 4.635, *p* = 0.006) and education (*F* = 6.716, *p* = 0.001) were significantly different between groups, that is, the participants in the TI-sham group were significantly younger and less educated than those in the other groups. In addition, a significant difference between groups was observed in both the accuracy (*F* = 3.613, *p* = 0.019) and the IES (*F* = 2.837, *p* = 0.047) of the 3-back test in the pre-stimulation period. We, therefore, performed analyses based on the percent changes in the outcome variables from pre- to during-stimulation and from pre- to post-stimulation and included age and education as covariates. It is worth noting that we conducted the one-way ANOVA with the corresponding pre-stimulation value as an additional covariate when accuracy and IES of the 3-back test revealed significant differences.

### Stimulation composed of two temporal interference electric fields validated by numerical calculation

**FIGURE 3:**
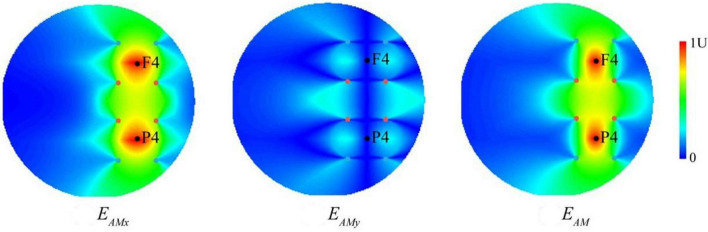
The computational modeling of temporal interference (TI). The envelope modulation amplitude distribution of temporally interfering electric field projected along *x*- and *y*-directions (EAMx and EAMy) as well as the combined electric field (EAM). The two black dots represent the F4 and P4 positions of the EEG cap, respectively, and the four sets of colored dots represent the electrode positions of two TI electric fields (i.e., two pairs of two alternating currents with the frequencies of 2,000 and 2,006 Hz), corresponding to those in Figure 2. Values are normalized to the peak.

To characterize the stimulation effect of two TI electric fields at a physical level, we modeled the spatial distribution of the envelope modulation amplitude (Figure 3). For each individual, the TI electric field on the upper or lower side and the envelope modulation amplitude distribution projected along *x*- and *y*-directions were consistent with the results of previous studies (Grossman et al., 2017). The current flow of our TI stimulation was of well focality, and the distribution of the envelope current was mainly within the target regions at the positions of F4 and P4. The amplitude distribution of the tACS and TI fields showed that (see Supplementary Figure 4) the two TI electric fields had a steeper spatial gradient compared to the tACS field, suggesting a more centralized hotspot. In addition, the maximum field amplitude in the hotspot of the isolated tACS field (see Supplementary Figure 4) was only half of the TI field when each electrode received the same current intensity. These results showed that substituting one electrode in an alternating current with a TI electric field leads to a more centralized stimulating hotspot, which is supposed to facilitate more precise and effective brain oscillation regulation. Our analyses were conducted using a cylindrical phantom, and the spatial distribution of the electrical field generated by TI was similar to that seen in previous studies using a spherical phantom or a head model (Lee et al., 2020). Our computational results focus on the determination of a geometric hotspot relative to the electrodes and the spatial gradient of the field amplitude as well as compare them to the results obtained from the isolated tACS field.

### Side effects

None of the participants reported serious side effects associated with TI stimulation. Only mild-to-moderate side effects/uncomfortable feelings were reported (Table 2). Kruskal–Wallis ANOVA models showed no significant difference in the number of participants who reported the side effects between the four groups (all *p* = 0.064–0.597). No participants reported skin redness in the TI or TI-sham groups. None of the participants in the tACS or tACS-sham groups reported a burning sensation; however, one participant in the TI group and two participants in the TI-sham groups reported a mild burning sensation. One participant in the tACS group reported moderate itchiness, but this was not reported by any of the participants in the other three groups. Only three participants reported fatigue after receiving TI, while six reported fatigue after receiving tACS. No other types of discomfort were reported by the participants.

**TABLE 2:** Reported side effects in the four types of stimulation (%).

	TI (*n* = 18)	tACS (*n* = 18)	TI-sham (*n* = 8)	tACS-sham (*n* = 10)	Mean of groups	*p*
**Pain**						
None	83.30% (15)	66.70% (12)	62.50% (5)	100.00% (10)	77.80%	0.139
Mild	11.10% (2)	33.30% (6)	12.50% (1)	0	16.70%	
Moderate	5.60% (1)	0	25.00% (2)	0	5.60%	
**Itchiness**						
None	72.20% (13)	83.30% (15)	100.00% (8)	90.00% (9)	83.30%	0.344
Mild	27.80% (5)	11.10% (2)	0	10.00% (1)	14.80%	
Moderate	0	5.60% (1)	0	0	1.90%	
**Burning**						
None	94.40% (17)	100.00% (18)	75.00% (6)	100.00% (10)	94.40%	0.064
Mild	5.60% (1)	0	25.00% (2)	0	5.60%	
Moderate	0	0	0	0	0	
**Skin redness**						
None	100.00% (18)	94.40% (17)	100.00% (8)	100.00% (10)	98.10%	0.572
Mild	0	5.60% (1)	0	0	1.90%	
Moderate	0	0	0	0	0	
**Fatigue**						
None	83.30% (15)	66.70% (12)	87.50% (7)	80.00% (8)	77.80%	0.597
Mild	11.10% (2)	27.80% (5)	12.50% (1)	10.00% (1)	16.70%	
Moderate	5.60% (1)	5.60% (1)	0	10.00% (1)	5.60%	

TI, temporal interference; tACS, transcranial alternating current stimulation. P-value represents the difference between groups.

### Blinding efficacy

Fisher’s exact test showed that the blinding was successful (*p* = 0.409), with only 5 of the 18 participants in the TI group guessing the stimulation condition correctly, which is not beyond the correct rate guessed by chance. Similarly, only 8 of the 18 participants in the tACS group guessed the stimulation condition correctly (see Supplementary Table 2 for details). The results showed that there was no correlation between the severity of the reported side effects and whether the participant was able to guess the type of stimulation they received (Kendall’s tau-b = −0.180–0.045, *p* = 0.166−725).

### The effects of temporal interference on working memory performance

The percent changes in accuracy, RT, and IES were used in the analysis due to the significant difference in these outcome variables between groups pre-stimulation (for details see Supplementary Table 1). The larger the positive percent changes observed in RT and IES, the better the improvement in WM. In contrast, the larger the negative percent change in the accuracy, the better improvement in WM.

#### Pre- to during-stimulation (percent change 1)

**FIGURE 4:**
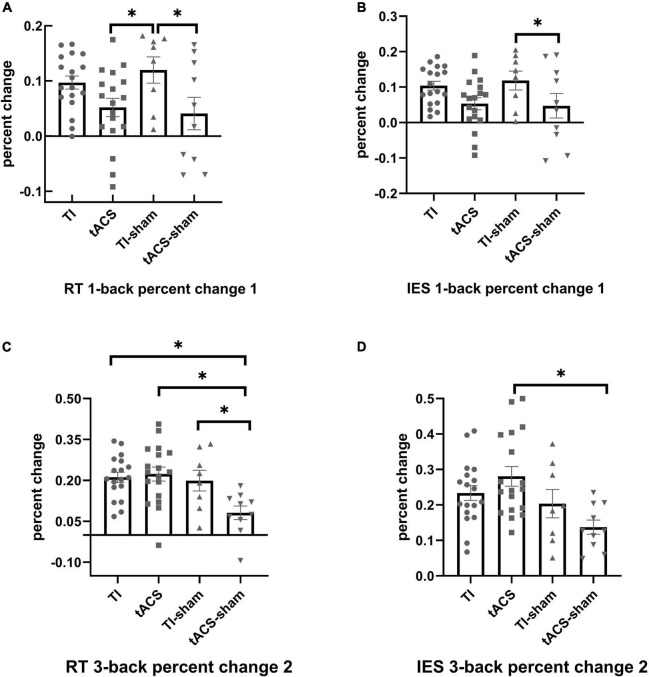
The effects of stimulation on the performance of N-back task. For RT, significantly greater percent changes were induced in the TI-sham group in the 1-back compared to tACS-sham and tACS (percent change 1) **(A)**; significantly greater percent changes were induced in TI, TI-sham, and tACS in the 3-back task compared to tACS-sham (percent change 2) **(C)**. For IES, significantly greater percent changes were induced in TI-sham in the 1-back task compared to tACS-sham (percent change 1) **(B)**; significantly greater percent changes were induced in tACS in the 3-back task compared to tACS-sham (percent change 2) **(D)**. Error bars represent SEM. *p < 0.05. The larger the positive percent change in RT and IES, the better the improvement in working memory. TI, temporal interference; tACS, transcranial alternating current stimulation; RT, reaction time; IES, inverse efficiency score. Percent change 1 = [(pre-stimulation values)–(during-stimulation values)]/(pre-stimulation values); Percent change 2 = [(pre-stimulation values)–(post-stimulation values)]/(pre-stimulation values); SEM, standard deviation.

For the primary outcome variable (i.e., those from 3-back task), no significant differences were found between the four stimulation groups for RT [*F*(3,48) = 2.380, *p* = 0.081], accuracy [*F*(3,47) = 0.720, *p* = 0.545], and IES [*F*(3,47) = 2.591, *p* = 0.064]. For the secondary outcome variables (1-back and 2-back), a significant difference was only observed in the 1-back test but not in the 2-back test (*F* < 0.670, *p* > 0.578) between the four stimulation groups. Furthermore, significant differences were found in the RT [*F*(3,48) = 5.053, *p* = 0.004] and IES [*F*(3,48) = 3.590, *p* = 0.020] measures of the 1-back task between the four stimulation groups. Education was a covariate that significantly affected the RT in the 1-back task (*p* = 0.035). Tukey’s *post-hoc* analysis showed that significantly greater reductions in the RT were induced in the TI-sham group compared to that in the tACS and tACS-sham groups (*p* < 0.05) (Figure 4A). For IES, Tukey’s *post-hoc* analysis revealed that the TI-sham group was significantly different (*p* < 0.05) compared to the tACS-sham group (Figure 4B).

#### Pre- to post-stimulation (percent change 2)

For the primary outcome variable, the ANOVA models showed that there were significant differences between the four stimulation groups in RT [*F*(3,48) = 5.826, *p* = 0.002] and IES [*F*(3,47) = 5.362, *p* = 0.003]. All covariates did not contribute to these differences. For RT, Tukey’s *post-hoc* analysis indicated that greater reductions were induced in the TI, TI-sham, and tACS groups compared to the tACS-sham group (all *p* < 0.05) (Figure 4C). The participants in the TI and tACS groups improved by an average of 171 and 203 ms from pre- to post-stimulation, respectively. For IES, Tukey’s *post-hoc* analysis showed that only the tACS group was significantly different (*p* < 0.05) from the tACS-sham group (Figure 4D). For the secondary outcome variables, no significant differences were observed between the four stimulation groups in the 1-back (*F* < 2.132, *p* > 0.101) and 2-back (*F* < 0.899, *p* > 0.449) tasks.

We further analyzed the effects of participant guesses regarding the stimulation conditions on the improvements induced by TI. The ANOVA models showed that within the TI group, no significant difference in the improvement of the mean RT was observed in the 3-back task between those who guessed their stimulation condition correctly and those who did not guess correctly (*p* = 0.64).

## Discussion

In this study, we examined the feasibility and safety of implementing TI stimulation in human participants and its effects on WM. In this study, it was observed that the TI stimulation technique we developed did not induce severe side effects/feelings of discomfort. The reported side effects were similar to those reported after receiving tACS as well as to those reported in the sham groups. This gives us confidence that TI stimulation is a safe NIBS technique that can be implemented in healthy younger adults. The blinding efficacy of TI stimulation was excellent, and there was no correlation between the severity of the side effects and the type of stimulation the participants guessed that they received. Using TI to target the frontoparietal regions only induced slight improvements in WM in the 3-back task when compared to tACS-sham (only in percent change 2), while no significant effects in any other outcomes, including TI and TI-sham, were observed. In addition, there seems to have been some improvement in WM in the TI-sham group. This pilot study provided critical knowledge regarding the application of TI for human participants, and studies with more rigorous design are highly demanded.

The actual mechanism of TI has yet to be fully understood. Some researchers have suggested that the TI effect may be due to the low-pass filtering property of the neural membrane, which prevents neural electrical activity from following very high-frequency oscillating (e.g., >1 kHz) electric fields (Hutcheon and Yarom, 2000; Grossman et al., 2017). It should be noted that passive low-pass filtering is not linear (because if it is a liner filter, no envelope signal can remain). Moreover, Mirzakhalili et al. (2020) proposed that if TI activated the deep regions of the brain, it may also produce multiple responses in tissues that are more superficial, which they called the “Sandwich” hypothesis. They argued that TI required an active ion-channel-mediated signal rectification process instead of passive membrane filtering. Therefore, future studies examining the effects of TI on the underlying neurophysiology of the brain are warranted.

No severe side effects were observed, and only mild-to-moderate side effects were reported after receiving TI stimulation, which is consistent with the previous TI experiment on human participants (Ma et al., 2021). The uncomfortable sensations induced by electrical stimulation are mainly related to the cutaneous receptor activity of the somatosensory system (Fertonani et al., 2015). Focused electrical stimulation activates fewer cutaneous receptors and thus reduces sensations of discomfort (Martinsen et al., 2004; Sheffield et al., 2022). The electric field simulation study also demonstrated that compared to tACS, TI was more centralized, with less co-stimulation of the cortical regions close to the electrodes (von Conta et al., 2021). This suggests that the skull skin making contact with the electrodes might be affected very little and that TI would not induce serious side effects. In future, we recommend that TI be validated further in other populations, such as in older adults or patients with neurological disorders.

Using tACS as a control condition was an advantage with regard to this study’s design. Although there were no significant differences between TI and tACS, our results may indicate that tACS appears to have an edge over TI in modulating WM in the 3-back task. Studies have shown that low-load cognitive tasks do not improve after active NIBS, regardless of whether carried out online or offline, which may be due to large ceiling effects at baseline (Hsu et al., 2015; Abellaneda-Pérez et al., 2019). In turn, WM performance improves at higher cognitive loads (Violante et al., 2017), which is consistent with the findings of our study. It should also be noted that though we used the same tasks and tACS montage, no significant improvements in 2-back task performance were observed as that was reported by Violante et al. (2017) in healthy participants. Neuroimaging studies using electroencephalography (EEG), magnetoencephalography (MEG), and functional MRI have demonstrated that WM is associated with the neural activities within the frontoparietal network (Wallis et al., 2015; Nielsen et al., 2017; Jones et al., 2020). The theta wave (4–8) of the brain activity organizes local neuronal ensembles across distant regions, which is related to WM processes (Rutishauser et al., 2010). However, our study did not use neuroimaging techniques to verify the specific mechanism producing the effect and did not eliminate the effects of high-frequency stimulation with an additional control group, and these need to be examined in future studies. In addition, the TI-sham showed greater improvement in N-back task performance compared to the tACS-sham or tACS groups. This unexpected result may be related to the large variance in the performance between individuals in a relatively small sample, suggesting that studies with larger sample sizes and more rigorous design should be conducted to further examine the effects of TI-sham.

This pilot study has some limitations. The sample size was relatively small, and only the effects of TI stimulation on functional/behavioral performance (i.e., WM) were assessed. Future studies using neuroimaging techniques (e.g., functional MRI) are needed to examine the underlying neurophysiological changes in the brain regions induced by TI. The questionnaire that was used to assess the side effects was not comprehensive and did not fully represent the subjective comfort level of the participants. In future, modified questionnaires should be applied to more accurately assess the side effects and subjective feelings after receiving electrical stimulation. Only the safety of receiving one session of TI was assessed; the potential issue of multiple repeated sessions of TI over days has not been examined. Current research has focused on the effects of a single TI stimulation during and immediately after stimulation. The effects of single stimulation duration and the potential effects of repeated stimulation need to be investigated further. We did not explore whether different stimulation durations would affect the results. In addition, this study was a single-blinded and randomized controlled study, and a double-blind and cross-over design would reduce the risk of observational bias and inter-individual variation. In this study, only a fixed frequency was pre-selected for stimulation; thus, further experiments are needed to verify whether other frequencies can induce functional improvements. In addition, the feasibility and safety of using TI to stimulate deep brain regions (e.g., basal ganglia) are worthy of examination in future studies. Nevertheless, the knowledge obtained from this study will help in the design of TI-based interventions in future studies examining its efficacy on important functions in humans.

## Data availability statement

The raw data supporting the conclusions of this article will be made available by the authors, without undue reservation.

## Ethics statement

The studies involving human participants were reviewed and approved by the Institutional Review Board of the Shanghai University of Sport. The participants provided their written informed consent to participate in this study.

## Author contributions

YZ, ZZ, JL, LL, and YL contributed to the study design. YZ and ZZ contributed to the data collection and analysis, hardware examination, and manuscript writing. ZQ and LL contributed to computation. JZ, JL, LL, and YL contributed to the revising of the manuscript. All authors contributed to the article and approved the submitted version.
