# Supplementary material for: Temporal interference stimulation targeting right frontoparietal areas enhances working memory in healthy individuals
Source: Front Hum Neurosci. 2022 Oct 28;16:918470. doi: 10.3389/fnhum.2022.918470 (PMC9650295; doi:10.3389/fnhum.2022.918470)
Supplement: Supplementary file 1 [file Data_Sheet_1.pdf]

Supplementary Materials

A circuit diagram of TIESS is shown in Supplementary Figure 1. Additionally, the system used in this study allowed for the simultaneous stimulation of two brain regions, whereas in Grossman's study, just one region was stimulated. Digital sinusoidal signals were generated by a custom program and were transformed to analog voltage signals using a digital-to-analog converter (USB-6361, National Instruments Inc., America). The voltage signals were delivered to the linear stimulus isolators (A395, World Precision Instruments Inc., America) to generate constant currents. The maximum values of the current intensity, voltage, and bandwidth of the isolators were 10 mA, 70 V, and 10 kHz, respectively.

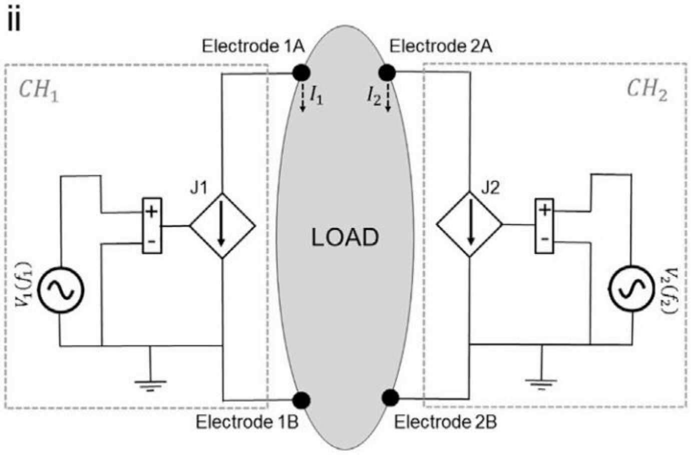

Supplementary Figure 1. Circuit diagram of TIESS (Grossman et al., 2017).

The stability of the current delivery in this TIESS was examined. According to Ohm's law, when the current is delivered constantly, the change in the voltage of the electric field is proportional to the change in the resistance in the electric circuit. Here, we compared this voltage–resistance relationship between TIESS and the Interferential Neuromodulation System (IFS, Soterix, USA), a commercialized tES product. The change in the voltage was measured using an oscilloscope. The current was set to have a peak-to-peak amplitude of 2 mA and a frequency of 2000 Hz. The initial resistance was 1KΩ and increased by 1KΩ during the test. The current intensity of TIESS remained stable and was compared to that in IFS across different resistance levels (Supplementary Figure 2). For example, when the resistance was 10KΩ, the current delivered by TIESS was 2.02mA, and the current delivered by the IFS was 1.94mA (Supplementary Figure 3).

Reference:

Grossman, N., Bono, D., Dedic, N., Kodandaramaiah, S. B., Rudenko, A., Suk, H. J., et al. (2017). Noninvasive deep brain stimulation via temporally interfering electric fields. Cell 169, 1029 – 1041.e16. doi: 10.1016/j.cell.2017.05.024

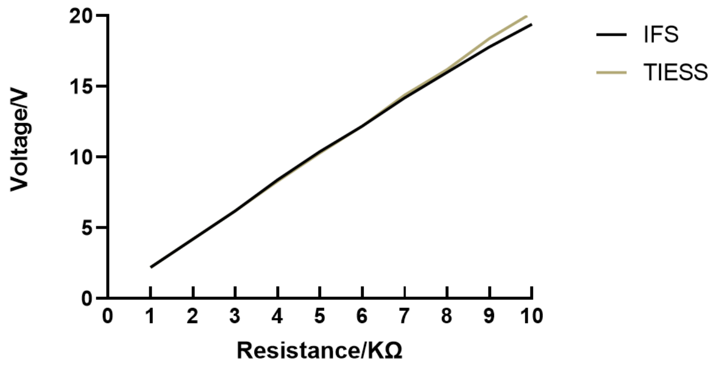

Supplementary Figure 2.

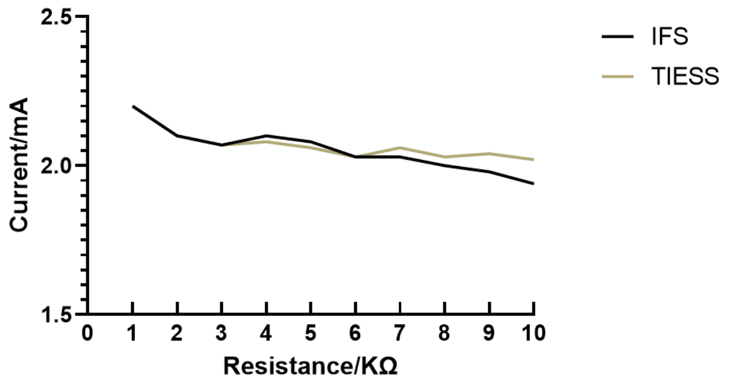

Supplementary Figure 3.

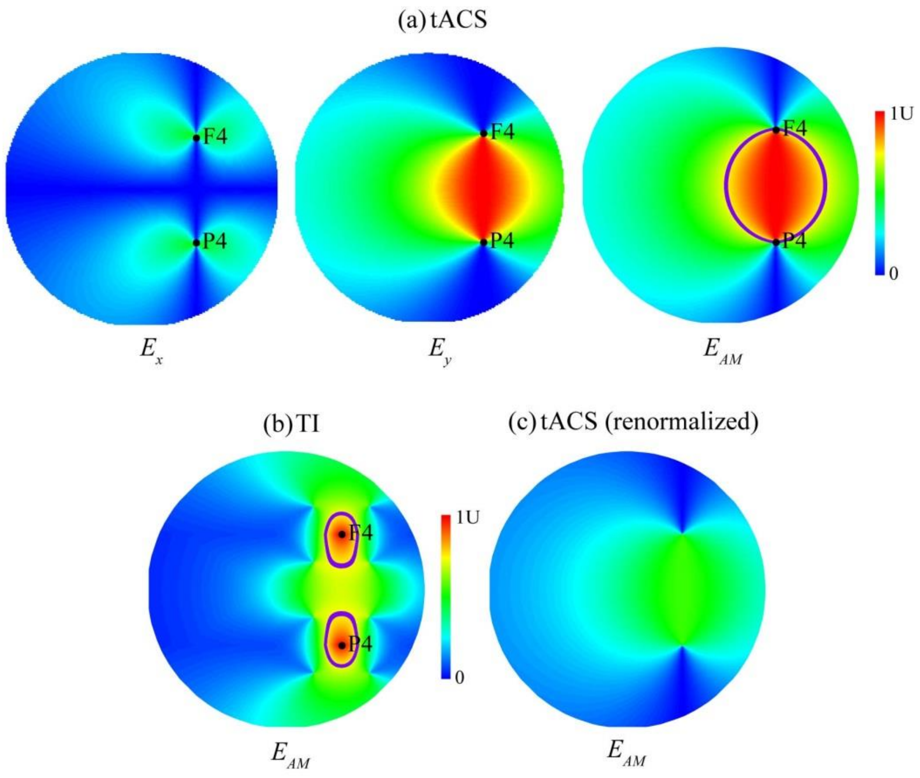

Supplementary Figure 4.

(a) The amplitude distribution of the tACS electric field (6 Hz) projected along the x- and y-directions as well as the combined electric field. (b) The envelope modulation amplitude distribution of the TI electric field. The two black dots represent the F4 and P4 positions of the EEG cap, respectively. Values are normalized to the peak in tACS or TI field. The 75% maximum contour line of the field amplitude is marked in purple. (c) The amplitude distribution of the tACS electric field with values renormalized to the peak in TI field. That is, the same color bar can be shared in insets b and c.

Supplementary Table 1. The percent change in the four groups (mean ± SD).

Variables	TI (n = 18)	tACS (n = 18)	TI-sham (n = 8)	tACS-sham (n = 10)
ACC 1-back percent change 1	-0.008 ± 0.026	-0.002 ± 0.024	0.001 ± 0.017	-0.009 ± 0.033
ACC 1-back percent change 2	-0.009 ± 0.023	0.010 ± 0.043	0.017 ± 0.042	-0.006 ± 0.043
RT 1-back percent change 1	0.097 ± 0.051	0.052 ± 0.070	0.120 ± 0.067	0.041 ± 0.093
RT 1-back percent change 2	0.122 ± 0.068	0.093 ± 0.077	0.161 ± 0.089	0.080 ± 0.075
IES 1-back percent change 1	0.104 ± 0.053	0.053 ± 0.072	0.118 ± 0.075	0.047 ± 0.110
IES 1-back percent change 2	0.129 ± 0.079	0.083 ± 0.083	0.144 ± 0.101	0.085 ± 0.088
ACC 2-back percent change 1	-0.018 ± 0.037	-0.028 ± 0.054	-0.002 ± 0.047	-0.026 ± 0.042
ACC 2-back percent change 2	-0.031 ± 0.031	-0.032 ± 0.071	0.008 ± 0.067	-0.035 ± 0.045
RT 2-back percent change 1	0.113 ± 0.075	0.126 ± 0.097	0.108 ± 0.086	0.121 ± 0.036
RT 2-back percent change 2	0.184 ± 0.092	0.169 ± 0.106	0.198 ± 0.115	0.183 ± 0.045
IES 2-back percent change 1	0.126 ± 0.091	0.147 ± 0.105	0.109 ± 0.093	0.142 ± 0.048
IES 2-back percent change 2	0.208 ± 0.095	0.193 ± 0.010	0.189 ± 0.120	0.209 ± 0.063
ACC 3-back percent change 1	-0.030 ± 0.057	-0.061 ± 0.102	-0.015 ± 0.042	-0.034 ± 0.063
ACC 3-back percent change 2	-0.032 ± 0.049	-0.089 ± 0.102	-0.009 ± 0.080	-0.067 ± 0.091
RT 3-back percent change 1	0.147 ± 0.072	0.136 ± 0.131	0.110 ± 0.136	0.045 ± 0.092
RT 3-back percent change 2	0.211 ± 0.079	0.223 ± 0.109	0.199 ± 0.108	0.081 ± 0.078
IES 3-back percent change 1	0.169 ± 0.086	0.181 ± 0.128	0.122 ± 0.133	0.076 ± 0.068
IES 3-back percent change 2	0.233 ± 0.090	0.280 ± 0.117	0.203 ± 0.113	0.137 ± 0.064

Abbreviations: 1-back, 2-back, 3-back: three types of N-back tests; TI: temporal interference; tACS: transcranial alternating current stimulation; ACC: accuracy; RT: reaction time; IES: inverse efficiency score. Percent change 1 = [(pre-stimulation values) - (during-stimulation values)] / (pre-stimulation values) and percent change 2 = [(pre-stimulation values) - (post-stimulation values)] / (pre-stimulation values). The larger the positive percent change in RT and IES, the better the improvement in working memory. In contrast, the larger the negative percent change in accuracy, the better the improvement in working memory.

Supplementary Table 2. Reported blinding efficacy in the four types of stimulation (%).

	TI (n = 18)	tACS (n = 18)	TI-sham (n = 8)	tACS-sham (n = 10)
Active stimulation	27.78% (5)	44.44% (8)	12.50% (1)	40.00% (4)
Sham stimulation	55.56% (10)	27.78% (5)	37.50% (3)	30.00% (3)
Stimulation unable to be identified	16.67% (3)	27.78% (5)	50.00% (4)	30.00% (3)

TI: temporal interference; tACS: transcranial alternating current stimulation.

Self-report Questionnaire

Name *

____

Gender *

____

Age *

____

Grade *

____

What kind of stimulation do you think you received today? *

○ Active stimulation

○ Sham stimulation

○ Cannot identify the stimulation

Side-effects feedback after the experiment *

Pain	☐0	☐1	☐2	☐3
Itching	☐0	☐1	☐2	☐3
Burning	☐0	☐1	☐2	☐3
Skin redness	☐0	☐1	☐2	☐3
Fatigue	☐0	☐1	☐2	☐3

Did you experience any other types of discomfort? If yes, please specify:

Note: severity of sensation rated as: none (0), mild (1), moderate (2) or severe (3).
